# Extending the Body to Virtual Tools Using a Robotic Surgical Interface: Evidence from the Crossmodal Congruency Task

**DOI:** 10.1371/journal.pone.0049473

**Published:** 2012-12-05

**Authors:** Ali Sengül, Michiel van Elk, Giulio Rognini, Jane Elizabeth Aspell, Hannes Bleuler, Olaf Blanke

**Affiliations:** 1 Center for Neuroprosthetics, School of Life Sciences, Ecole Polytechnique Fédérale de Lausanne (EPFL), Lausanne, Switzerland; 2 Laboratory of Cognitive Neuroscience, Brain Mind Institute, Ecole Polytechnique Fédérale de Lausanne (EPFL), Lausanne, Switzerland; 3 Systems Robotic Laboratory, Ecole Polytechnique Fédérale de Lausanne (EPFL), Lausanne, Switzerland; 4 Department of Neurology, University Hospital of Geneva (HUG), Geneva, Switzerland; University of Reading, United Kingdom

## Abstract

The effects of real-world tool use on body or space representations are relatively well established in cognitive neuroscience. Several studies have shown, for example, that active tool use results in a facilitated integration of multisensory information in peripersonal space, i.e. the space directly surrounding the body. However, it remains unknown to what extent similar mechanisms apply to the use of virtual-robotic tools, such as those used in the field of surgical robotics, in which a surgeon may use bimanual haptic interfaces to control a surgery robot at a remote location. This paper presents two experiments in which participants used a haptic handle, originally designed for a commercial surgery robot, to control a virtual tool. The integration of multisensory information related to the virtual-robotic tool was assessed by means of the crossmodal congruency task, in which subjects responded to tactile vibrations applied to their fingers while ignoring visual distractors superimposed on the tip of the virtual-robotic tool. Our results show that active virtual-robotic tool use changes the spatial modulation of the crossmodal congruency effects, comparable to changes in the representation of peripersonal space observed during real-world tool use. Moreover, when the virtual-robotic tools were held in a crossed position, the visual distractors interfered strongly with tactile stimuli that was connected with the hand via the tool, reflecting a remapping of peripersonal space. Such remapping was not only observed when the virtual-robotic tools were actively used (Experiment 1), but also when passively held the tools (Experiment 2). The present study extends earlier findings on the extension of peripersonal space from physical and pointing tools to virtual-robotic tools using techniques from haptics and virtual reality. We discuss our data with respect to learning and human factors in the field of surgical robotics and discuss the use of new technologies in the field of cognitive neuroscience.

## Introduction

Complex tool-use is a uniquely human activity and its achievement enabled a remarkable step forward in the evolution of our species. In daily life we use spoons, knives, forks, pencils, rulers and scissors. When enjoying sports we use a variety of equipment such as golf clubs or tennis rackets. In addition, we often interact with tools to control computers, such as a keyboard, computer mouse and joysticks. In a certain sense, even vehicles can be considered as tools that greatly extend the boundaries of our physical body and our bodily capabilities [1], [2]. Thus, tools can be used to extend our action space and to perform many tasks of daily life, whether at home, at work, or for recreation [3], [4].

To perform common daily tasks easily, we use different tools that can be categorized based on their function and characteristics. Holmes and Spence [5] classified tools into three categories: physical interaction tools, pointing tools and detached tools. Physical interaction tools function as a physical connection between the body and environment (e.g. a brush, a stick, a pen etc.). They are often hand-held objects that are purposefully used to interact with other objects to achieve a goal (e.g. brushing the floor). Many studies have described the changes in the neural representation of multisensory peripersonal space with physical tools in healthy adults [6]–[8]. The second category consists of pointing tools that are typically used to point at another object (e.g. a laser pointer). There is no direct physical connection between the user and the objects with which they interact. Some studies have investigated the changes in the neural representation of multisensory peripersonal space with pointing tools in healthy participants [9], [10]. The last category of tools defined by Holmes and Spence [5] are detached tools. A human operator uses an interface to perform a task at distant locations or even in virtual reality (computer screen). With detached tools there is no direct physical linkage between the user and the target whereas those tools exhibit force and motion coupling between the human and virtual environment. Up to now, the effect of detached tool use on the neural representation of multisensory peripersonal space has not been studied.

Recent advances in robotics have brought attention to a specific class of detached tools: human-robot interfaces. Robotic tools, such as surgery robots and telemanipulators are designed to operate at distant locations or in virtual scenarios under direct human control [11], [12]. These unprecedented tools greatly increase the precision, force and accessibility of human manipulation (within the human body or within industrial systems such as piping, turbines etc.). These tools allow increased dexterity by down-scaling position or up-scaling forces. Such devices are also called ‘master-slave’ systems: they have a master side, which detects the positions and motion of the user and sends this information to a slave robot that is in contact with the remote environment [13]. Surgeons now frequently and efficiently interact with such telemanipulators to perform complex tasks, such as laparoscopic surgery [14]–[16].

Despite these technical advances in the field of surgical robots and telemanipulators, only little attention has been paid to the psychological and cognitive mechanisms that underlie the interactions with these devices. The aim of these robots is to increase the telepresence and transparency between the surgeon and the environment, as well as the accuracy and intuitiveness of the use of the system. The precise role of the different factors that contribute to the extension of one's body and one's peripersonal space during virtual tool use remains unknown. For instance, it is unclear whether the inclusion of sensory modalities, such as touch or haptic feedback, would result in a more realistic interaction and thereby improve the control over the robot. In order to address these questions we here propose a cognitive neuroscience approach to robotics. More specifically, we measured the multisensory integration of vision and touch when operating a haptic device and evaluated the potential of such newly emerging surgical robotic devices at the level of their perception as a tool by the human brain [17].

The integration of information from different sensory modalities by the human brain is a complex process that has received a lot of attention in cognitive neuroscience. Previous studies have shown for instance, that the space around the body (near extrapersonal space or peripersonal space) is represented in the brain differently than the extrapersonal space that is far from the body [18]. Peripersonal space representation is based on the multisensory integration of visual, tactile, somatosensory, and auditory cues in the frontal and parietal lobes of the human cortex [18]. The brain representation of peripersonal space is highly plastic and has been shown to adapt dramatically to physical tool use, for example by extending or projecting peripersonal space beyond its normal range to also include the end of a handheld tool. Several studies using single unit recordings in macaque monkeys and the spatial modulations of multisensory behavior in healthy participants and brain-damaged patients have supported the idea that tool use extends multisensory peripersonal space [19]–[21]. One method for studying the integration of multisensory information in relation to tool use is the crossmodal congruency task [21], [22]. In this task, participants are required to respond to the elevation of tactile stimuli to the thumb and index finger, while at the same time ignoring visual distractors presented at the end of the tool. Participants are asked to make speeded elevation judgments (up vs down) to the vibrotactile stimulus while ignoring the visual distractors. The crossmodal congruency effect (CCE) is defined as the reaction time difference between incongruent conditions (light and vibration in opposite position, i.e. up vs. down) and congruent conditions (light and vibration both up or both down). The CCE is considered a reliable measure of multisensory integration in peripersonal space, as it has been shown that the CCE is enhanced for objects that can be easily integrated in the body representation, such as rubber hands and virtual bodies [21], [22]. CCEs have been shown to reflect changes in hand ownership [23], [24] as well as full-body ownership [25]. Recently, CCEs have been used to study the effect of tool-use and in particular whether humans experienced changes in the representation of peripersonal space. It has been found, for instance, that after active tool use, visual distractors presented at the tip of the tool interfered with tactile stimuli presented at the hand of the participant holding the tool, suggesting that the peripersonal space representation was changed with tool use [8], [21], [22] and that peripersonal space was extended by active tool use [8].

Although several tool-use studies have now established these changes in the representation of peripersonal space by using the CCE, they were all done with simple physical tools such as golf clubs, rakes or sticks. Advanced robotic tools such as haptic devices or master/slave systems have to our knowledge not yet been investigated in this context (but see Moizumi et al., 2007). Compared to physical tools, these advanced tools behave like ‘tele-arms’ and user movements from the master side are mapped on the remote tools. For instance, in the case of a surgical robotic system, the slave robot performs the movements of the surgeon who can be working at a different physical location. One of the aims in surgical robotics is to increase the immersion and telepresence of the surgeon into the remote site by using appropriate display and telepresence technologies to regain virtually direct access to the operation - comparable to the experience during open surgery. Hence, the study of the neurocognitive aspects of these advance tools is important from an engineering perspective. For instance, studying how the peripersonal space representation changes with virtual robotic tool use would provide an objective evaluation of the telepresence and the usability of the surgical robotic systems. This will give new insights in the design of usable and immersive surgical robotic system that can easily be integrated by the brain and are experienced by the surgeons as a ‘tele-arm’. In addition, virtual tools enable to study the different factors involved in the remapping of peripersonal space (e.g. providing haptic feedback, changing the mapping from the arm movements to the tool movements etc.; cf. Sengül et al. in prep.). The present study provides the first step towards investigating virtual tool use using cognitive neuroscience methods.

We tested whether the peripersonal space representation would change ‘naturally’ even in the case of a telemanipulated tool, i.e. without a mechanical connection between the hand (master side) and the point of physical action (slave side). As a benchmark, the well-known experiment of Maravita et al. [8], performed with physical tools (golf clubs), was reproduced here in a new technological context (multimodal haptic interface operating on a VR physical scene). The participant's task was to cross or uncross the virtual golf clubs in experiment 1 and to just hold the tool interface in experiment 2, in which the crossing and uncrossing was done by the experimenter. Subsequently, participants were required to make speeded elevation discriminations of vibrotactile stimuli delivered to the thumb and index finger – using a foot pedal while ignoring visual distractors presented at the end of the virtual tool. Unlike physical tools, virtual robotic tools behave like ‘tele-arms’. User movements from the master side are mapped on the remote tools. In combination with appropriate displays (3D monitors or head mounted displays) and telepresence technologies (realistic 3D graphics), they aim to enable a high level of immersion into the remote site. Because of these different physical and information processing mechanism in virtual-robotic tools compared to physical tools, we hypothesize that the neural representation of the peripersonal space would change quickly due to the immersive nature of these tools. In the first study we tested whether the active use of virtual-robotic tools would alter the spatial dependency of CCEs in a similar way as found in Maravita's (2002) experiment with physical tools [8]. That is, we expected that visual distractors would interfere with tactile stimuli applied to the hand that is holding the tool and this should be the case not only for the uncrossed posture but also for the crossed posture. In the second study we tested whether the spatial modulation of the CCE according to the tool posture (i.e. crossed or uncrossed) is the result of the active use of the tools or not. To this end in the second experiment the tools were not crossed actively, but passively by the experimenter. We expected an interference of visual distractors with the tactile stimuli connected to the hand by the tool for both the uncrossed posture and the crossed posture due to the immersive and ‘tele-arm’-like nature of the virtual robotic tools, thereby facilitating a remapping of peripersonal space even when the tool is only used passively.

## Materials and Methods

### Subjects

A total of 19 healthy right-handed participants took part in these experiments: Ten participants (2 female, ages 21–24, mean age (SE): 22.3 (1.2) years) in study 1. Nine participants (3 female, ages 19–28, mean age (SE) 23.2 (2.6) years) in study 2. All participants had normal or corrected to normal vision, no disorder of touch and had no history of neurological or psychiatric conditions. Each experiment took approximately 60 minutes per participant. The participants were informed about the general purpose of the research, were fully debriefed and were given the opportunity to ask questions and to comment on the research after the experiment. All participants gave written informed consent and were compensated for their participation. The experimental procedure was approved by the local research ethics committee – La Commission d'éthique de la recherche Clinique de la Faculté de Biologie et de Médecine – at the University of Lausanne, Switzerland and was performed in accordance with the ethical standards laid down in the Declaration of Helsinki.

### Materials and Apparatus

**Figure 1:**
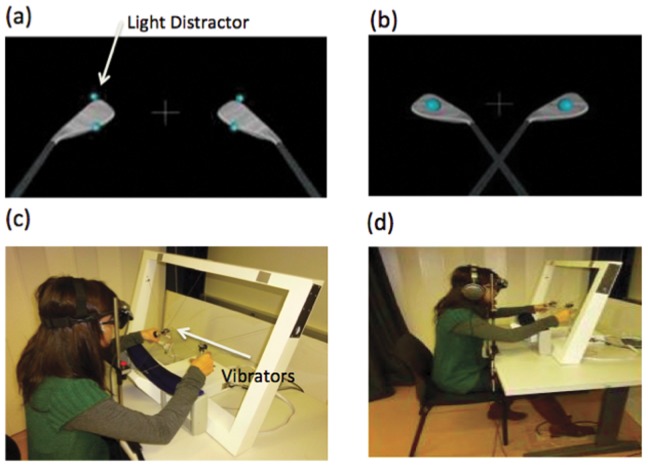
Virtual reality views and experimental setup used in the experiments. (A) Virtual tools in an uncrossed posture: the small balls on the upper and lower part of the tools are the visual distractors. They are presented simultaneously with the vibrotactile stimuli to distract the participants. They can be presented at the same positions as the vibrotactile stimuli (congruent) or at different positions (incongruent). (B) Virtual tools in a crossed posture: The big balls in the middle of the tools have two functions. First they indicate that the CCE phase is finished. Second they indicate the position to locate the tools to keep the distance between each tool constant. The big cross in the middle of the tools is the fixation point. (C, D) A cable driven haptic device (the Da Vinci Simulator) with a large workspace was used. Participants interacted with the virtual object through the handles of the device. Their interactions were shown through a head mounted display. To mask the noise of the vibrators and environmental noise, headphones were used to present white noise. Participants responded to vibrotactile stimuli using the foot pedal. A chin rest system was used to prevent undesired movement of the head. The participant has given written informed consent (according to the PLoS guidelines) for the publication of her picture.

We employed a robotic system consisting of a bimanual haptic interface for the training of operations with the da Vinci surgery system (Mimic's dV-Trainer™, Mimic Technologies Inc., Seattle USA [26] see Figure 1D). The da Vinci system is a well-known surgical robotic system that is used for minimally invasive surgical procedures. This novel device is the first test-bed for the telerobotic surgical simulator. The tracking of the hand movements and force feedback are provided through a cable-driven system. Since it is a cable driven system, it has a large workspace. It provides 7 Degrees of Freedom (DOF) in motion for each hand and it can render high forces in 3-translation directions (x,y,z) without instability. The system has two lightweight grippers that enable transparent interactions with virtual reality making realistic bimanual manipulations possible. The participants were seated at a table and held two haptic interfaces, one in their left and one in their right hand. The index and thumb of both hands were positioned in the haptic device as shown in Figure 1D and their movements and interactions with virtual objects were presented on a head mounted display (HMD, eMagin Z800 3DVisor, 1.44 megapixel resolution).

An open source platform, CHAI 3D, and a set of C++ libraries were used for the modeling and for simulating the haptics, and for visualization of the virtual world. This platform supports several commercial haptic devices and it is possible to extend it to support new custom force feedback devices. We have extended this platform by adding the drivers and libraries of our custom force feedback device.

Two vibrotactile target stimulators (Precision MicroDrive shaftless vibration motors, model 312-101, 3 V, 80 mA, 9000 rpm (150 Hz) 1.7 g with a diameter of 12 mm and a length of 3.4 mm) were attached to the participants' thumb and index finger. Foam and rubber padding was used to insulate the vibrotactile stimulators from the surrounding material, thus minimizing any conduction of vibrations through the haptic device itself. For each participant, these stimulators were tested to generate easily localizable and clearly perceptible sensations. Vibrotactile stimuli were driven by electrical signals generated by a desktop computer (Intel Core i7 CPU with 2.8 GHz, 3 GB or Ram, with NVIDIA GeForce 9800 GT Graphic Card). Two data acquisition cards (NI PCI-6014 and NI PCI 6052E) were used to detect pedal responses and to drive vibrotactile stimulators. To minimize any unwanted reflections, the participants were seated in a dimly illuminated room enclosed by black curtains.

The participants viewed two virtual-robotic tools through a head mounted display. The distance between the tools subtended approximately 35° of visual angle. Visual distractor stimuli subtended approximately 0.9° of visual angle, positioned at the upper and lower locations of tips of the virtual-robotic tools that had a visual angle of 6.4°. For the modeling, simulating the haptics, and visualization of the virtual world, CHAI 3D and a set of C++ libraries were used. A virtual world with two virtual golf clubs was developed, see Figure 1 A and B.

A fixation cross was positioned at the vertical and horizontal mid-point of the corresponding four LEDs on the two tools. A chin rest system was used to prevent undesired movement of the head. In order to measure the participant's response, two pedals were attached to the floor next to the participant's right foot. The pedal separation was adjusted to fit the participant's foot size. One of the pedals was placed under the heel and the other under the toe of the participant's right foot. The participant raised his toes to indicate that the vibrations were felt at the index finger or raised his heels to indicate that the vibrations were felt at the thumb. White noise was presented over the headphones at an adequate level so that participants could not hear the sound of the vibrotactile stimulators or the operation of the other hardware during the experiments.

### Experimental Design and Procedure

The experiments were designed in a 2×2×2 factorial manner. The 3 within-participants factors were congruency of the elevation of the vibrotactile stimuli with respect to the visual distractors (congruent vs. incongruent), the vibrotactile target side relative to the visual distractor side (same vs. different), and the type of posture (uncrossed vs. crossed). There were two blocks of 16 practice trials each, which were not analyzed. Experimental blocks began when the participant achieved an accuracy of more than 85 percent. A total of 480 experimental trials were given, divided into 15 blocks, with 240 trials for the straight tools and 240 trials for the crossed tools. Participants actively crossed or uncrossed the tools between every four trials in study 1 and changed passively (tool crossing made by the experimenter rather than the participants by crossing the tools only visually on the screen) at the end of 240 trials in study 2. Each of the 16 conditions (4 visual distractors×4 vibrotactile target locations) was presented 15 times (crossed or uncrossed), in a pseudo-randomized order determined by the computer.

Participants sat in front of a table and the table height was adjusted for each participant. They held the two haptic interfaces, with their thumbs next to the lower vibrotactile stimulator and their index fingers next to the upper stimulator. They were instructed to make speeded elevation discriminations to the vibrotactile stimuli. They were told that visual distractors would be presented simultaneously with the vibrotactile stimuli but that they should ignore them as much as possible while they were responding. They were instructed not to close their eyes and fixate on the central fixation cross until the end of the trial. The participant's right foot rested on the two pedals. They were instructed to hold both pedals pressed, which was the default condition and to release the toes in response to tactile stimuli applied to the index finger or to depress the heel if the stimuli were applied to the thumb. This toe/heel response mapped to index/thumb to make it compatible with upper/lower elevation of the vibrotactile and visual stimuli. In each trial, the visual distractor stimulus was presented 100 ms before presentation of the vibrotactile stimulus (SOA 100 ms) as previous work showed that this maximizes the CCE [27].

Participants moved two virtual golf clubs via the handle of a bimanual haptic simulator. In the first study, for some trials they held two tools in a straight position and for some trials they actively crossed the tools. Tool posture was changed actively after every four CCE trials. After every four CCE trials, to indicate that the CCE task was finished, two light balls were shown. This instructed the participant to cross or to uncross the tools. These two light balls also functioned to indicate where to position the tools in order to keep the distance between the tools constant for the crossed and uncrossed posture. In the second study the tools were not changed actively after every four CCE trials but only changed at the end of 240 trials. Virtual robotic tools were crossed by the experimenter rather than the participants as in the Maravita et al.'s experiment with physical tools [8] and only visual feedback of the crossed golf clubs was presented.

### Analysis

For CCE analysis, trials with an incorrect response were discarded from the RT analysis but they were analyzed in the percentage error analysis. Trials with RTs larger than 1.500 and less than 200 milliseconds were removed. Next, RT outliers were removed using a criterion of 3 standard deviations above or below the subject's mean RT. These led to a rejection of a mean ± SE of 4.9±0.45% of all trials in experiment 1, and 6.7±0.65% of all trials in experiment 2. Data from all trials that resulted in correct responses were analyzed by using a repeated-measures three-way analysis of variance (ANOVAs) on the mean values of RTs. The three factors in the ANOVA design were: Congruency (congruent/incongruent), Side (same/different) and Tool posture (uncrossed/crossed). Paired t-tests were used for post-hoc comparisons on the CCEs. In addition, following previous studies on the CCE, the inverse efficiency (IE) was calculated by dividing the reaction time by the accuracy (proportion correct) for each condition, thereby controlling effectively for any speed-accuracy trade-off in the reaction time data [22].

## Results

### Study 1: Active tool use

**Figure 2:**
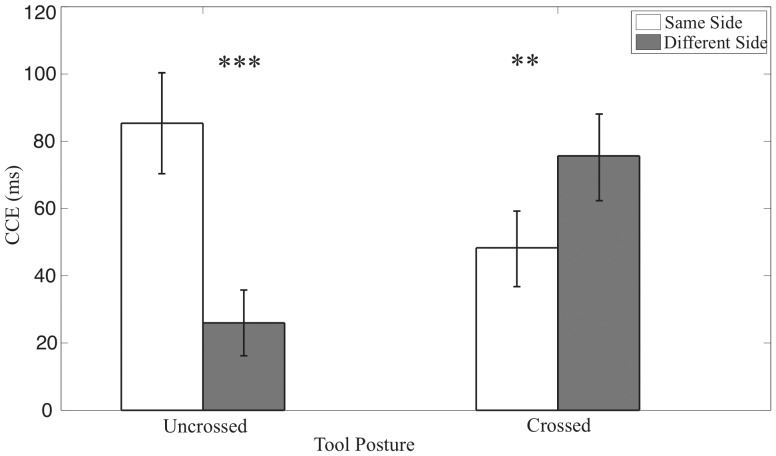
Crossmodal congruency effect (CCE) with standard error in Experiment 1. The CCE was calculated as incongruent reaction times minus congruent reaction times. White bars represent the condition in which visual stimuli were presented to the same visual hemifield with tactile stimuli, black bars represent trials in which the visual stimuli were presented to the different hemifield. The bars on the left side are for the uncrossed posture and bars on the right side are for the crossed posture.

Congruency effects derived from RT data from the first experiment are represented in Figure 2 and Table 1. The ANOVA performed on RTs from Experiment 1 revealed a main effect of congruency (F (1, 9) = 32.90, p<0.001) and a significant interaction between congruency and side (F (1, 9) = 9.07), p<.05) confirming that CCEs were significantly larger in the same side conditions compared to the different side conditions (t(9) = 3.02; p<.05). Crucially, we also found a three-way interaction between Congruency, Side and Tool Posture (F (1, 9) = 37.88, p<0.001).

**Table 1:** Mean reaction times (RT) in milliseconds, percentage of errors (%) and inverse efficiency (IE) for Experiment 1.

Experiment 1			
Same Side			
Tool Posture	Congruent	Incongruent	Mean CCE
RT	682.4(26.2)	730.8(21.7)	48.3(10.9)
Crossed %	1.43(0.63)	1.59(0.49)	0.16(0.71)
IE	691.6(26.3)	741.3(19.2)	49.7(11.8)
RT	678.8(25.9)	764.1(32.5)	85.4(15.0)
Uncrossed %	0.94(0.49)	4.57(0.97)	3.63(1.12)
IE	686.1(27.3)	804.2(41.1)	118.1(20.7)

Different Side			
Tool Posture	Congruent	Incongruent	Mean CCE
RT	673.5(26.3)	749.2(27.1)	75.6(12.5)
Crossed %	1.32(0.58)	4.10(1.00)	2.78(0.91)
IE	682.3(25.7)	786.4(34.0)	104.2(17.3)
RT	718.7(27.1)	744.7(27.6)	26.0(9.8)
Uncrossed %	1.41(0.57)	2.91(0.70)	1.50(0.67)
IE	728.7(28.0)	763.8(28.2)	35.1(12.5)

The left column represents data for congruent conditions, the middle column for incongruent conditions. The right column represents the crossmodal congruency effect (CCE; i.e. difference between incongruent and congruent conditions). The first rows represent data for the crossed tool posture and the second rows represent data for the uncrossed posture. The upper panel represents data for visual stimuli at the same side as the tactile vibrations, the lower panel represents data for visual stimuli at the different side compared to the tactile vibrations. Values in parentheses are standard errors.

To determine the driving factor of this three-way interaction, we performed post-hoc comparisons between the same side CCE versus the different side CCE for the RT measures for each tool posture. This analysis revealed that the CCE differed significantly between same side and different side for the uncrossed (t(9) = 5.80; p<0.001) and crossed condition (t(9) = 3.30; p<0.01) (see Figure 2 and Table 1). As inspection of Figure 2 reveals, the direction of these effects differed for uncrossed versus crossed conditions. In line with data obtained with physical tools, for the uncrossed condition the same side CCEs were larger than the different side CCEs, whereas for the crossed condition the different side CCEs were larger than the same side CCEs. These data indicate that active tool use results in a remapping of peripersonal space, depending on the position of the tools.

The ANOVA on the error rates revealed a main effect of congruency (F(1, 9) = 21.75, p<0.01) and a tendency for a three-way interaction between Congruency, Side and Tool Posture (F (1, 9) = 3.97, p = 0.077). The ANOVA on the IE data revealed a main effect of congruency (F(1, 9) = 40.21, p<0.001 and a significant interaction between congruency and side (F (1, 9) = 5.52), p<.05) and a three-way interaction between Congruency, Side and Tool Posture (F (1, 9) = 14.99, p<0.01) (see Table 1). Thus the analysis of the IE confirms the main findings from the analysis of Reaction Times and Error rates, indicating that the CCE side effect is modulated by the posture of the virtual tools (i.e. crossed or uncrossed). More importantly, this analysis provides further support that this effect cannot be accounted for by a speed-accuracy trade-off between the different experimental conditions.

### Study 2: Passive tool use

**Figure 3:**
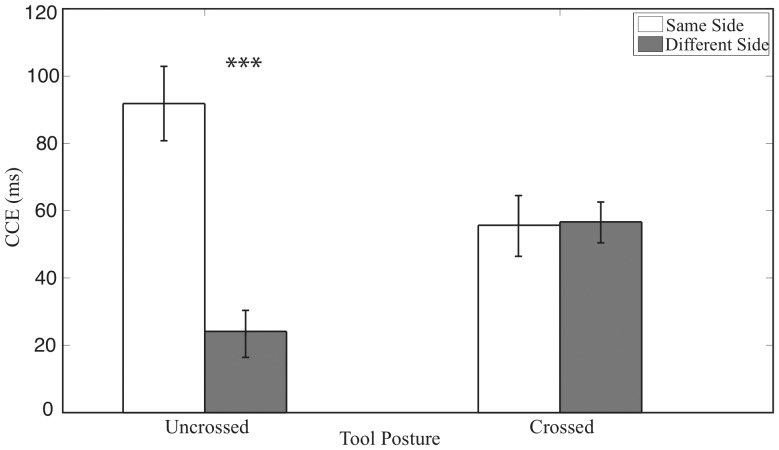
Crossmodal congruency effect (CCE) with standard error in Experiment 2. The CCE was calculated as incongruent reaction times minus congruent reaction times. White bars represent the condition in which visual stimuli were presented to the same visual hemifield with tactile stimuli, black bars represent trials in which the visual stimuli were presented to the different hemifield. The bars on the left side are for the uncrossed posture and on bars on the right side are for the crossed posture.

Reaction time data from the second experiment are represented in Figure 3 and Table 2. The ANOVA performed on RTs from Experiment 2 revealed a main effect of tool posture (F (1, 8) = 5.37, p<0.05), a main effect of congruency (F (1, 8) = 142.00, p<0.001) and a significant interaction between side and congruency (F (1, 8) = 17.63), p<0.005) reflected in a stronger same side CCE than a different side CCE (t(8) = 4.6; p<0.005). Crucially, we found a three-way interaction between Congruency, Side and Tool Posture (F (1, 8) = 12.18, p<0.01). We also performed post-hoc comparisons between the same side CCE versus the different side CCE for the RT. This analysis revealed that the CCE difference between same side and different side was significant for the uncrossed (t(8) = 4.58; p<0.01) but not significant for the crossed case (t(8) = 0.11; p = 0.91 NS)(see Figure 3 and Table 2). As can be seen in Figure 3, same side CCEs were larger than the different side CCEs only for the uncrossed condition but not for the crossed conditions. This finding indicates that passive tool use did not result in a complete remapping of peripersonal space, according to the position of the tools.

**Table 2:** Mean reaction times (RT) in milliseconds, percentage of errors (%) and inverse efficiency (IE) for Experiment 2.

Experiment 2			
Same Side			
Tool Posture	Congruent	Incongruent	Mean CCE
RT	601.0(19.7)	656.7(22.6)	55.7(8.8)
Crossed %	1.33 (0.55)	3.79 (1.02)	2.46 (1.22)
IE	609.2(20.6)	681.8(21.7)	72.7(4.5)
RT	591.7(18.0)	683.5(24.8)	91.8(11.1)
Uncrossed %	2.63 (0.92)	9.62 (0.99)	6.99 (1.13)
IE	607.7(19.2)	754.4(26.4)	146.7(14.6)

Different Side			
Tool Posture	Congruent	Incongruent	Mean CCE
RT	595.5(22.6)	652.2(23.7)	56.7(5.9)
Crossed %	2.67 (0.72)	6.24(1.79)	3.57(2.23)
IE	624.2(28.4)	683.6(25.7)	59.4(22.1)
RT	643.9(24.1)	668.0(21.6)	24.1(6.3)
Uncrossed %	4.16(1.12)	3.55(1.28)	−0.61(1.22)
IE	671.1(24.1)	692.8(22.4)	21.7(10.8)

The left column represents data for congruent conditions, the middle column for incongruent conditions. The right column represents the crossmodal congruency effect (CCE; i.e. difference between incongruent and congruent conditions). The first rows represent data for the crossed tool posture, the second rows represent data for the uncrossed posture. The upper panel represents data for visual stimuli at the same side as the tactile vibrations, the lower panel represents data for visual stimuli at the different side compared to the tactile vibrations. Values in parentheses are standard errors.

The ANOVA performed on error rates revealed a main effect of congruency (F (1, 8) = 21.21, p<0.01), a main effect of tool posture (F (1, 8) = 5.42, p<0.05) and an interaction between tool posture and congruency (F (1, 8) = 13.36, p<0.01). We also found a three- way interaction between Congruency, Side and Tool Posture for the percentage of errors (F (1, 8) = 5.78, p<0.05). We performed post-hoc comparisons between same side CCE versus different side CCE for the error rates. This analysis revealed that the difference between same side and different side was significant for the uncrossed posture (p<0.01) but not significant for the crossed posture. The ANOVA performed on the IE data revealed a main effect of tool posture (F (1, 8) = 17.70, p<0.01), a main effect of congruency, (F (1, 8) = 78.71, p<0.001) and an interaction between side and congruency (F (1, 8) = 30.28, p<0.01). We also found a three-way interaction between Congruency, Side and Tool Posture (F (1, 8) = 10.87, p<0.05) (see Table 2). Thus, the ANOVA on the IE data confirms the main findings of the analysis of the RT and error data and provides further support that no speed-accuracy trade-off underlies the present results.

In experiment 1, we found a significant three-way interaction between Congruency, Side, and Tool posture, as expected. In experiment 2, this interaction was also significant. In order to directly investigate the difference between active and passive tool crossing, we performed a between-experiments comparison, using a 4-way ANOVA with Experiment as a between-participants variable. The 4-way ANOVA performed on RTs revealed a main effect of tool posture (F (1, 17) = 5.01, p<0.05), a main effect of congruency (F (1, 17) = 97.38, p<0.001), a significant interaction between side and congruency (F (1, 17) = 27.67), p<0.001) and a three-way interaction between Congruency, Side and Tool Posture (F (1, 17) = 42.52, p<0.001). The 4-way interaction was not significant (F (1, 17) = 0.57, p = 0.46 NS) suggesting a remapping according to tool posture for both active and passive tool crossing.

In addition, to explore the pattern of interference reversal statistically, we analyzed the interference effect by pairs of blocks [8]. We could not find any significant correlation of the same side distractors or different side distractors with block number for the straight or crossed tools. Only a tendency for a negative correlation of same side distractors with the block number for the active straight tool was observed (r = −0. 86 p = 0.059). The result of this analysis suggests that there is no learning effect of remapping. This analysis provides further evidence that the remapping of peripersonal space using virtual tools happens instantaneously.

## Discussion

In the present study we investigated the integration of visuo-tactile cues in the case of a multimodal robotic interface controlling a virtual-robotic tool. At least three findings support the notion that the use of such ‘virtual-robotic’ tools facilitates the integration of multisensory information in peripersonal space. First, our results show that there was an interaction of vision and touch as reflected in the crossmodal congruency effect (CCE) for virtual robotic tools. Second, it was found that actively crossing the tool resulted in a remapping of peripersonal space, as reflected in a stronger CCE when visual stimuli appeared at a different side than the tactile vibration, at the tip of the tool that was held in the stimulated hand. Third, it was found that this remapping of peripersonal space did not depend on active tool use, as passive crossing of the tools resulted in a change in the CCE side effect as well. These results therefore extend previous findings on visuo-tactile integration in tool-use [20], [22], [26] to the domain of virtual tools, haptic interfaces and surgical robotics.

First, the results of both experiments showed an interference effect of visual distractors presented on the virtual-robotic tools on tactile discrimination judgments. The interfering effect of the visual stimuli on the virtual-robotic tools was reflected in slower reaction times and increased error rates when participants responded to incongruent compared to congruent vibrotactile stimuli, which is known as the crossmodal congruency effect (CCE). The results of the first experiment showed that visual distractors presented at the end of the left tool interfered more strongly with judging tactile stimuli applied to the left hand compared to the right hand (and vice versa for visual distractors presented at the right tool). Therefore, these findings suggest that both the passive and the active use of a virtual-robotic tool can alter multisensory integration in peripersonal space, reflecting a remapping of peripersonal space similar to the effects found for active physical and pointing tool use [6], [8], [9], [18]. Thus, when the participants used the virtual-robotic tools actively, our data suggest that they functioned as an extension of their arms. This extension produced a stronger association between the vibrotactile stimuli on the hands and the visual stimulation at the end of the tool. The effect of visual distractors on tactile discrimination responses has often been related to findings in monkeys, indicating that the response properties of neurons in parietal areas reflect the functional aspects of tool use (i.e. incorporation of the tool in the body; cf. [10], [19]; but for critical discussion, see: [22]). A similar neural mechanism has been proposed to underlie the effects of multisensory integration after tool use, as seen in healthy humans as well as brain damaged patients [21]. For instance, Farne el al. [28] studied the extension of peripersonal space with physical tool-use in visuo-tactile neglect patients and found that visuo-tactile extinction can be modulated by tool use (i.e. stronger left tactile extinction with right lights when a tool is wielded on the right side).

Second, in the first experiment it was found that when the virtual-robotic tools were actively crossed, visual distractors from the opposite visual field interfered more strongly with tactile stimuli applied to the hand that was holding the tool. In the crossed condition the tip of the tool held by the left hand was in the right visual field and visual distractors presented at the tip of this tool interfered more strongly with tactile stimuli applied to the left compared to the right hand. The opposite was true for the tool held by the right hand. Thus, visual stimuli were primarily associated with the hand that was holding the tool rather than the spatial side at which the stimuli appeared. This finding suggests that actively crossing the tool resulted in a remapping of peripersonal space, as reflected in a stronger CCE when visual stimuli appeared at a different side than the tactile vibration, at the tip of the tool that was held in the stimulated hand. The finding that active crossing of the virtual-robotic tool resulted in a remapping of peripersonal space extends previous studies on real-world tools [8], [19], [20]. These studies showed that crossing the tools actively remapped the visuo-tactile representation of peripersonal space [8].

The results of the second experiment, obtained in a different participant sample, showed that when participants held uncrossed tools, the CCE was larger when the visuotactile stimuli were presented at the same side compared to the different side. In contrast to the results obtained in the first experiment, when the tools were passively crossed, the CCEs for visuotactile stimuli presented at the same and different side were comparable in size. Thus, the passive crossing of the virtual-robotic tools did not completely remap the peripersonal space representation, as it did in the first experiment. It did affect the representation of peripersonal space, as reflected in the fact that the difference between the same and different side CCE differed between crossed and uncrossed postures (i.e. a significant 3-way interaction was found). This result is different to that in previous studies (see [8]), in which passively moving the real-world tool always resulted in a stronger CCE for visuotactile stimuli appearing at the same compared to the different side, irrespective of whether the tools were crossed or not. Our findings suggest that virtual-robotic tools may alter multisensory integration even when the tool is not actively used and thus affect multisensory integration differently than the physical tools. This could also be due to different physical and information processing mechanism of the virtual robotic tools such as the immersive nature and ‘tele-arm’ like behavior of these tools. In our experiments the real hands were not visible, as the participants were wearing head mounted displays. The absence of visual information about the hands may have facilitated the integration of the virtual tools in the body representation, thereby resulting in a remapping of the visuo-tactile representation of peripersonal space even in the case of passive tool use.

The results of this study are novel and extend previous studies for several reasons. Up to now, studies on the representation of peripersonal space in humans have used mainly tools that physically link peripersonal space and extrapersonal space - such as golf clubs, rakes, long sticks to press a button or to reach a distant object [6], [8]. It has been found, for instance, that peripersonal space was extended by active tool use [8]. However, there is an alternative interpretation with respect to the extension of peripersonal space with the tool. Holmes et al. have shown that multisensory spatial interactions were enhanced at the tips of the tools rather than in the middle of tools, suggesting that peripersonal space is not extended but projected towards the part of the tool that is most relevant for the task. According to this interpretation, tools act as spatial attentional cues rather than entities which cause an extension of peripersonal space [29]. In fact, virtual robotic tools can help to shed light on the question of whether peripersonal space is projected or extended, by enabling novel experimental paradigms with virtual robotic tools that would be difficult to perform with physical tools. For instance, in a recent study we inserted a movable joint in the middle of the tool, thereby making the middle part of the tool more relevant to the action at hand (e.g. as if the middle part of the tool represents one's wrist or elbow; cf. Sengül et al. in prep.). It was found that peripersonal space was selectively projected towards the part of the tool that was relevant to the task at hand (i.e. controlling the tool by moving the wrist or the elbow), thereby providing further support for the idea that peripersonal space is indeed projected to distant locations that are task-relevant and attended.

The results of the present study also extend previous studies with pointing tools [9], [10]. For instance, Iriki et al (2001; cf. [10]) conducted a study in which monkeys trained to control a tool via a computer screen with the arms out of view. It was found that the visual receptive field size of visuo-tactile neurons in parietal areas was enlarged to include the tool viewed through the video monitor. Interestingly, when only a cursor was presented instead of a tool, much fewer neurons with such properties were found. It could be that for monkeys it is more difficult to integrate abstract visual information into their body representation. In contrast, for humans the interaction with virtual objects is omnipresent in our everyday lives (e.g. the use of computers, video games, PC tablets, etc.) and as a consequence virtual tools may be more easily integrated in the representation of our body. In support of this view, Bassolino et al. showed that the space where a pointing tool (i.e. a computer mouse) was actually held (i.e. close to hand) was extended to the space where it operates (i.e. the computer screen) even though these spaces were not physically connected [9].

Furthermore, the findings of this study are in line with the findings of Moizumi et al. [30], reporting a remapping of touch in VR with humans holding the arms in a crossed or uncrossed position. In this study a temporal order judgment task was used and it was found that when the arms were uncrossed participants' ability to report tactile vibrations applied to the hand was modulated by whether the virtual tools were crossed or uncrossed. In contrast, when the arms were crossed, the direction of the force feedback primarily determined the perceived order of tactile judgments, indicating the importance of haptic force feedback for disambiguating the referral of tactile sensations. The present study extends these findings [30], by showing that virtual tool use changes visuo-tactile instead of only tactile interactions in virtual space. In addition, our findings indicate that both active crossing and passive crossing of the virtual tool results in a remapping of the peripersonal space. Finally, we would like to point out that we investigated peripersonal space representations, using a new class of virtual tools that are increasingly used in surgical robotics. In surgical robotics, an important aim is to increase the telepresence of the surgeon in the remote site. Studying how peripersonal space representation as measured through the CCE changes with virtual robotic tools could be an objective assessment method for the evaluation of the telepresence by analysing whether the remote site was represented as within peripersonal space or not.

We note that the present work on the relation between the CCE and peripersonal space representations, is also consistent with the findings by Rognini et al., who showed that visuo-tactile CCEs can be obtained in a robotically mediated environment using virtual hands [26]. The present study, however, shows that visuo-tactile integration on a robotic platform does not only occur for virtual hands but also for virtual-robotic tools. Moreover, Rognini et al. measured the integration of visuo-tactile cues online: during the holding and moving of virtual objects with virtual hands. In the present study, we measured visuo-tactile CCEs after using a telemanipulation tool. Hence we propose that measuring such CCE post-effects can also be used as an objective assessment of how we learn to use robotic tools.

Together, the results of these studies show that cognitive neuroscience measures can be used to investigate the integration of visual and tactile cues in robotic technologies. This suggests that CCE measurements may be used as an objective assessment of human factors. Up to now, human factors in robotics, especially in surgical robotics was quantified by means of questionnaires or performance based measurements such as task completion time or task accuracy [31]–[33]. These studies focused on a specific task such as needle insertion during surgical procedures, neglecting more basic and repeated behavioral changes of multisensory integration driven by tool-use [34]–[36]. Here we propose a new methodology to study ‘human factors’ in a surgical interface with a more objective assessment technique by quantifying visuo-tactile integration measured by the CCE. This extends and changes the standard analysis of human factors in the field of surgical robotics by injecting insights and methods from cognitive neuroscience into this emerging field between neuroscience, psychology, and engineering. Understanding how the brain codes peripersonal space and which factors contribute to the integration of tools into the brain's body representation may turn out to be of pivotal importance for the design of future robots that can be more easily used and controlled for the benefit of patients nearby and at distance. For instance, an important issue in the field of medical and surgical robotics is the feeling that the surgeon is holding, and operating with a surgical tool – as if he were controlling a real tool to operate his patient. The present study provides a first step to study more objectively the relation between real tools and virtual-robotic tools, by showing that similar neurocognitive mechanisms are involved in using real and virtual-robotic tools.

In summary, the present paper presented two experiments on multisensory integration through the use of a virtual-robotic tool. It was found that virtual-robotic tool use changed the integration of visuo-tactile information in peripersonal space, as reflected in a cross-modal congruency effect (CCE). This result illustrates that, in order to change the representation of peripersonal space, it is not necessary to have a physical connection between the space where the tool is held and the space where the tool operates. The results are consistent with previous studies on the cross-modal congruency effect (CCE). This study establishes that telemanipulators consisting of haptic devices and virtual reality can be used in cognitive neuroscience investigations, thereby opening up exciting new possibilities for neuroscience experimentation and improved incorporation of human factors into the future design of minimally invasive surgical robots.
